# Association between duration of antimicrobial prophylaxis and postoperative outcomes after lumbar spine surgery

**DOI:** 10.1017/ice.2021.529

**Published:** 2022-12

**Authors:** Mary W. Porter, William Burdi, Jonathan D. Casavant, McKenna C. Eastment, Luis G. Tulloch-Palomino

**Affiliations:** 1 Pharmacy Services, VA Puget Sound Health Care System, Seattle, Washington; 2 Hospital and Specialty Medicine, VA Puget Sound Health Care System, Seattle, Washington; 3 Division of Allergy and Infectious Diseases, Department of Medicine, University of Washington School of Medicine, Seattle, Washington

## Abstract

**Objectives:**

To describe the association between duration of antimicrobial prophylaxis (AMP) and 30-day surgical site infection (SSI), 7-day acute kidney injury (AKI), 90-day *Clostridioides difficile* infection (CDI), prolonged hospitalization, and 30-day reoperation after lumbar spine surgery for noninfectious indications, and to report adherence to current guidelines.

**Design:**

Survey.

**Participants and setting:**

The study cohort comprised 6,198 patients who underwent lumbar spine surgery for noninfectious indications across 137 Veterans’ Health Administration surgery centers between 2016 and 2020.

**Methods:**

Used univariate and multivariate logistic regression to determine the association between type and duration of AMP with 30-day SSI, 7-day AKI, 90-day CDI, prolonged hospitalization, and 30-day reoperation.

**Results:**

Only 1,160 participants (18.7%) received the recommended duration of AMP. On multivariate analysis, the use of multiple prophylactic antimicrobials was associated with increased odds of 90-day CDI (adjusted odds ratio [aOR], 5.5; 95% confidence interval [CI], 1.1–28.2) and 30-day reoperation (aOR, 2.3; 95% CI, 1.2–4.4). Courses of antimicrobials ≥3 days were associated with increased odds of prolonged hospitalization (aOR,1.8; 95% CI, 1.4–2.3) and 30-day reoperation (aOR, 3.5; 95% CI, 2.2–5.7). In univariate analysis, increasing days of AMP was associated with a trend toward increasing odds of 90-day CDI (cOR, 1.4; 95% CI, 1.0–1.8 per additional day; *P* = .056).

**Conclusions:**

Longer courses of AMP after lumbar spine surgery were associated with higher odds of CDI, prolonged hospitalization, and reoperation, but not with lower odds of SSI. However, adherence to the recommended duration of AMP is very low, hinting at a wide evidence-to-practice gap that needs to be addressed by spine surgeons and antimicrobial stewardship programs.

Surgical site infections (SSIs) complicate ∼3% of spine surgeries.^1^ Antimicrobial prophylaxis (AMP) has been shown to decrease SSIs after spine surgery, but the duration of AMP remains a frequent subject of debate between antimicrobial stewardship programs (ASPs) and spine surgeons.

To date, most of the available evidence has not shown a difference in the frequency of SSIs after short and long courses of prophylactic antimicrobials, but some studies have yielded conflicting results.^5–9^ Consequently, despite recommendations to limit prophylactic antimicrobials to a single dose or a single day, in practice, the duration of prophylactic therapy ranges from 1 to 10 days,^2–4^ and many patients receive antibiotic courses that may be unnecessarily long and lead to increased acute kidney injury (AKI), *Clostridioides difficile* infection (CDI), and healthcare costs.^10,11^

Our primary aim was to determine the association between the duration of AMP and 30-day SSI, 7-day AKI, 90-day CDI, prolonged hospital length of stay (LOS), and 30-day reoperation after lumbar spine surgery for noninfectious indications. Additional aims included determining the effect of antimicrobial selection on these outcomes and describing the degree of adherence to guidelines regarding selection and duration of prophylactic antimicrobials.

## Methods

### Data sources and study population

The Veterans’ Health Administration (VHA) is the largest integrated healthcare system in the United States, offering surgical care at 109 inpatient and 28 ambulatory surgery centers.^12^ For this study, we matched perioperative variables from the Veterans’ Affairs Surgical Quality Improvement Program (VASQIP) database to bar-code medication administration (BCMA) records from a database created by the VA Informatics and Computing Infrastructure (VINCI) team. The VASQIP uses a validated sampling algorithm to manually review 105 perioperative variables from ∼70% of all major surgical cases performed within the VHA.^13^ The database created by VINCI is a relational database that contains detailed clinical, pharmacy, and laboratory data abstracted directly from the VHA integrated electronic health record (EHR). We included all veterans from the VASQIP database who underwent lumbar spine surgery (primary CPT-4 codes 22224, 22533, 22534, 22558, 22587, 22612, 22630, 22633, 22862, 22865, 63005, 63017, 63012, 63030, 63042, 63047) between January 1, 2016, and September 30, 2020, and received at least 1 day of AMP with cefazolin, clindamycin, and/or vancomycin at any time starting on the date of surgery up to, and including, 7 days after the date of surgery. For patients who underwent multiple spine surgeries within the study period, we included only the first episode of care in this analysis. We excluded individuals who received other antibiotics and/or had postoperative *International Classification of Disease, Tenth Revision* (ICD-10) codes for epidural abscess (G06., G07.), osteomyelitis, discitis, other infectious spondylopathy (M46.1, M46.2, M46.3, M46.4, M46.5), or infection and inflammatory reaction due to prosthetic material (T84.5, T84.6).

### Antimicrobial prophylaxis

Individuals were categorized by type (cefazolin, clindamycin, vancomycin, and multiple antimicrobials) and duration (1, 2, 3, and ≥4 days) of prophylactic antimicrobials. The duration of therapy was defined as the total sum of days for which any amount of cefazolin, clindamycin, and/or vancomycin was administered between the date of surgery and 7 days after the date of surgery.^14^ If a patient received >1 of the specified prophylactic antimicrobials, their duration of therapy was the sum of the days of therapy of all administered agents.

### Outcomes

The primary outcome of interest was 30-day SSI, which was defined as the development of superficial, deep, or organ-space SSI in the 30 days after the date of surgery. The VASQIP reviewer used the National Healthcare Safety Network (NHSN) definitions to categorize the different SSI categories.^15^ We defined 7-day AKI according to Acute Kidney Injury Network (AKIN) criteria (postoperative serum creatinine increase of ≥0.3 mg per dL or ≥50% of the preoperative value) or as determined by the VASQIP reviewer.^16^ Only 1,155 patients had enough data for the 7-day AKI outcome. The 90-day CDI outcome was defined as the presence of a positive *C. difficile* laboratory result in the 90 days following the date of surgery. Prolonged hospital length of stay (LOS) was defined as an LOS greater than the mean LOS for the entire cohort (4 days). The 30-day reoperation outcome was defined as a return to the operating room for any reason in the 30 days after the date of surgery.

### Covariates

Covariates were selected prior to data collection based on published risk factors for our outcomes of interest. These included demographic details (age, sex, race, ethnicity), comorbid conditions (ie, body mass index [BMI] in m/kg^2^, history of diabetes mellitus, history of hypertension, American Society of Anesthesiologists (ASA) classification), substance or medication use (ie, tobacco use <1 year prior to the surgery date, corticosteroid use <30 days prior to the surgery date), surgical details (ie, surgery type, anesthesia type, duration of surgery, heavy blood loss defined as blood loss requiring ≥4 units of packed red blood cells (pRBCs) in the 3 days after the date of surgery), and postoperative infections.^1,10,17^ Postoperative sepsis was defined as documentation of sepsis in the EHR by the primary physician or the presence of infection plus ≥2 of the following in the immediate postoperative period: temperature >38°C or <36°C, heart rate >90 beats per minute, respiratory rate >20 breaths per minute, PaCO2 <32 mmHg, leukocyte count >12,000 or <4,000 cells per mm^3^, or >10% immature (band) forms with or without hypotension. Postoperative pneumonia and urinary tract infection (UTI) were defined according to NHSN criteria.^15^

### Statistical analysis

Clinical and surgical characteristics were summarized for the entire cohort and by duration of prophylactic antimicrobials. We used univariate and multivariate logistic regression to test the association between different types and durations of AMP with our outcomes of interest (30-day SSI, 7-day AKI, 90-day CDI, prolonged hospital LOS, and 30-day reoperation). Our multivariate logistic regression model adjusted for age, BMI, diabetes mellitus, hypertension, ASA classification, tobacco use, corticosteroid use, surgery type, duration of surgery, and antimicrobial type or duration. For the 7-day AKI, 90-day CDI, and prolonged hospital LOS outcomes, we also adjusted for other postoperative infections (sepsis, pneumonia, UTI). Lastly, we stratified our analysis of the impact of duration on these outcomes by selected antimicrobial agent. Analyses were conducted in Stata version 15.1 software (StataCorp, College Station, TX). All tests were 2-sided, and *P* < .05 was considered statistically significant.

### Ethical statement

The study was approved by the institutional review board of the Veterans’ Affairs Puget Sound Healthcare System. The requirement for informed consent was waived.

## Results

We included 6,198 veterans who underwent lumbar spine surgery: 2,041 (32.9%) underwent arthrodesis or fusion, 858 (13.8%) underwent discectomy, and 3,299 (53.2%) underwent laminectomy. Of all participants, 5,384 (86.9%) received the recommended agent (cefazolin), but only 1,160 (18.7%) received the recommended duration (≤1 day) of prophylactic antimicrobials.

The average age of this patient cohort was 60.9 years (SD ±12.6), 5,748 (92.7%) were men, 4,543 (73.3%) were classified as ASA class 3, and 2,010 (32.4%) had used tobacco <1 year before surgery. The average duration of surgery was 3.1 hours (SD ±1.7).

The rate of 30-day SSI was 1.3% (n = 83), including 44 (0.7%) superficial SSIs, 30 (0.5%) deep SSIs, and 9 (0.1%) organ-space SSIs. The rate of 7-day AKI was 7.1% (n = 82 of 1,155); the rate of 90-day CDI was 0.3% (n = 18), and the rate of 30-day reoperation was 3.6% (n = 223). The average hospital LOS was 3.9 days (SD ±9.4) (Table 1).

**Table 1.** Clinical and Surgical Characteristics of 6,198 Veterans Included in this Analysis from January 1, 2016 through September 30, 2020

Variable	Total (N = 6,198)		1 Day (N = 1,160)		2 Days (N = 4,163)		3 Days (N = 381)		≥4 Days (N = 494)		*P* Value
	%	No.	%	No.	%	No.	%	No.	%	No.	
**Demographics**											
Age, mean y (SD)	60.9 (12.6)	6,198	61.3 (12.6)	1,160	60.4 (12.6)	4,163	62.2 (11.9)	381	63.1 (11.9)	494	<.001
Sex, male	92.7	5,748	92.5	1,073	92.5	3,851	95.0	362	93.5	462	.282
**Race** ^**a**^											
White	74.3	4,606	75.2	872	74.7	3,109	73.2	279	71.9	355	.198
Black	17.0	1,054	18.7	217	16.6	693	18.1	69	15.2	75	.235
Other	2.4	148	1.6	19	2.3	96	3.7	14	3.8	19	.017
Ethnicity^b^											
Hispanic or Latino	5.5	343	4.8	56	5.5	230	7.1	27	6.1	30	.371
**Comorbidities**											
BMI, mean kg/m^2^ (SD)^c^	30.4 (5.7)	6,196	30.4 (5.4)	1,160	30.5 (5.9)	4,162	30.3 (4.9)	380	30.4 (5.2)	494	.983
Diabetes mellitus	30.2	1,874	29.2	339	30.0	1,250	32.0	122	33.0	163	.390
Hypertension	69.8	4,327	71.1	825	69.1	2,876	70.6	269	72.3	357	.319
Tobacco	32.4	2,010	31.0	360	33.7	1,403	28.9	110	27.7	137	.010
Corticosteroids	2.3	41	2.7	31	2.2	91	2.1	8	2.2	11	.794
**ASA Class**											
1	0.8	52	0.7	8	1.0	41	0	0	0.6	3	.175
2	22.6	1,398	20.8	241	23.9	997	22.3	85	15.2	75	<.001
3	73.3	4,543	75.3	873	72.0	2,997	74.3	283	78.9	390	.003
4	3.3	205	3.3	38	3.1	128	3.4	13	5.2	26	.085
**Surgery details**											
Arthrodesis/Fusion	32.9	2,041	33.3	386	31.9	1,327	29.7	113	43.5	215	<.001
Discectomy	13.8	858	14.9	173	13.1	544	17.1	65	15.4	76	.057
Laminectomy/Laminotomy	53.2	3,299	51.8	601	55.1	2,292	53.3	203	41.1	203	<.001
Duration of surgery (mean, std), hours	3.1 (1.7)	6,198	3.1 (1.8)	1,160	3.1 (1.5)	4,163	3.1 (1.6)	381	3.7 (2.2)	494	<.001
Required ≥4 pRBCs	0	2	0	1	0	1	0	0	0	0	.549
**Antimicrobial details**											
Duration of AMP (mean, std), days	2.1, 1.0	6,198	…		…		…		...		…
Cefazolin	86.9	5,384	77.9	904	89.8	3,737	83.2	317	86.2	426	<.001
Clindamycin	4.1	256	4.7	55	4.4	184	1.6	6	2.2	11	.005
Vancomycin	7.5	462	16.8	195	4.9	204	10.5	40	4.7	23	<.001
Multiple^d^	1.5	96	0.5	6	0.9	38	4.7	18	6.9	34	<.001
**Postoperative infections**											
Sepsis	0.5	29	0.7	8	0.4	15	0.3	1	1.0	5	.111
Pneumonia	0.1	8	0	0	0.1	5	0	0	0.6	3	.043
UTI	0.5	33	0.5	6	0.5	22	0.3	1	0.8	4	.736
**Postoperative outcomes**											
30-day SSI	1.3	83	1.6	19	1.3	56	0.8	3	1.0	5	.560
Superficial SSI	0.7	44	0.9	10	0.7	29	0.5	2	0.6	3	.916
Deep SSI	0.5	30	0.5	6	0.5	22	0.3	1	0.2	1	.863
Organ space SSI	0.1	9	0.3	3	0.1	5	0	0	0.2	1	.527
7-day AKI^e^	7.1	82/1,155	7.9	15/190	6.4	48/746	9.8	9/92	7.9	10/127	.614
90-day CDI	0.3	18	0.3	4	0.2	9	0.5	2	0.6	3	.183
Hospital LOS, mean d (SD)^f^	3.9, 9.4	5,812	4.2 (15.5)	1,093	3.5 (7.7)	3,851	4.8 (5.5)	378	6.3 (5.0)	490	<.001
30-day reoperation	3.6	223	3.0	35	2.3	94	10.0	38	11.3	56	<.001

Note. SD, standard deviation; BMI, body mass index; ASA, American Society of Anesthesiologists; pRBCs, packed red blood cells; AMP, antimicrobial prophylaxis; UTI, urinary tract infection; SSI, surgical site infection; AKI, acute kidney injury; CDI, *Clostridioides difficile* infection; LOS, length of stay.

a 390 (6.3%) participants declined to answer or did not report race.

b 116 (2.6%) participants declined to answer or did not report ethnicity.

c BMI results were available for 6196 participants.

d Any combination of cefazolin, clindamycin, and vancomycin.

e 7-day acute kidney injury results were available for 1,155 participants.

f Hospital LOS results were available for 5,812 participants.

The use of multiple antimicrobials increased the odds of 90-day CDI (crude odds ratio [cOR], 8.2; 95% CI, 1.8–36.4; adjusted odds ratio [aOR], 5.5; 95% CI, 1.1–28.2) and 30-day reoperation (cOR, 4.9; 95% CI, 2.7–8.7; aOR, 2.3; 95% CI, 1.2–4.4). Additionally, patients who received >1 agent also had a higher rate of prolonged hospital LOS in univariate analysis only (cOR, 3.1; 95% CI, 2.0–4.7) (Table 2).

**Table 2.** Crude and Adjusted Odds Ratios for Postoperative Outcomes Based on Type of Antimicrobial Prophylaxis

Postoperative outcomes^a,b^	Crude Odds Ratio (95% CI)			Adjusted Odds Ratio (95% CI)		
	Clindamycin	Vancomycin	Multiple	Clindamycin	Vancomycin	Multiple
30-day SSI	2.0 (0.9–4.7)	**2.0 (1.1–3.9)**	1.8 (0.4–7.3)	2.1 (0.9–4.9)	1.8 (0.9–3.5)	2.3 (0.5–10.0)
7-day AKI^c^	0.4 (0.1–1.7)	0.9 (0.3–2.2)	0.8 (0.1–6.4)	0.4 (0.8–1.6)	0.5 (0.2–1.4)	0.8 (0.9–6.2)
90-day CDI^c^	1.5 (0.2–11.5)	0.8 (0.1–6.3)	**8.2 (1.8–36.4)**	1.5 (0.2–12.1)	0.6 (0.8–5.2)	**5.5 (1.1–28.2)**
Prolonged hospital LOS^c^	1.0 (0.7–1.3)	1.2 (1.0–1.5)	**3.1 (2.0–4.7)**	1.1 (0.8–1.4)	1.0 (0.8–1.3)	1.4 (0.9–2.3)
30-day reoperation	0.6 (0.2–1.4)	1.4 (0.9–2.1)	**4.9 (2.7–8.7)**	0.6 (0.3–1.6)	1.2 (0.7–1.9)	**2.3 (1.2–4.4)**

Note. CI, confidence interval; SSI, surgical site infection; AKI, acute kidney injury; CDI, *Clostridioides difficile* infection; LOS, length of stay; UTI, urinary tract infection; *P* < 0.5 for values in bold.

a Reference group is cefazolin.

b All multivariate models adjusted for age, BMI, diabetes mellitus, hypertension, ASA classification, tobacco use, corticosteroid use, surgery type, duration of surgery, and duration of prophylactic antimicrobials (1, 2, 3, and ≥4 days).

c The multivariate models for 7-day AKI, 90-day CDI, and prolonged hospital LOS are also adjusted for other postoperative infections (sepsis, pneumonia, UTI).

**Fig. 1.**
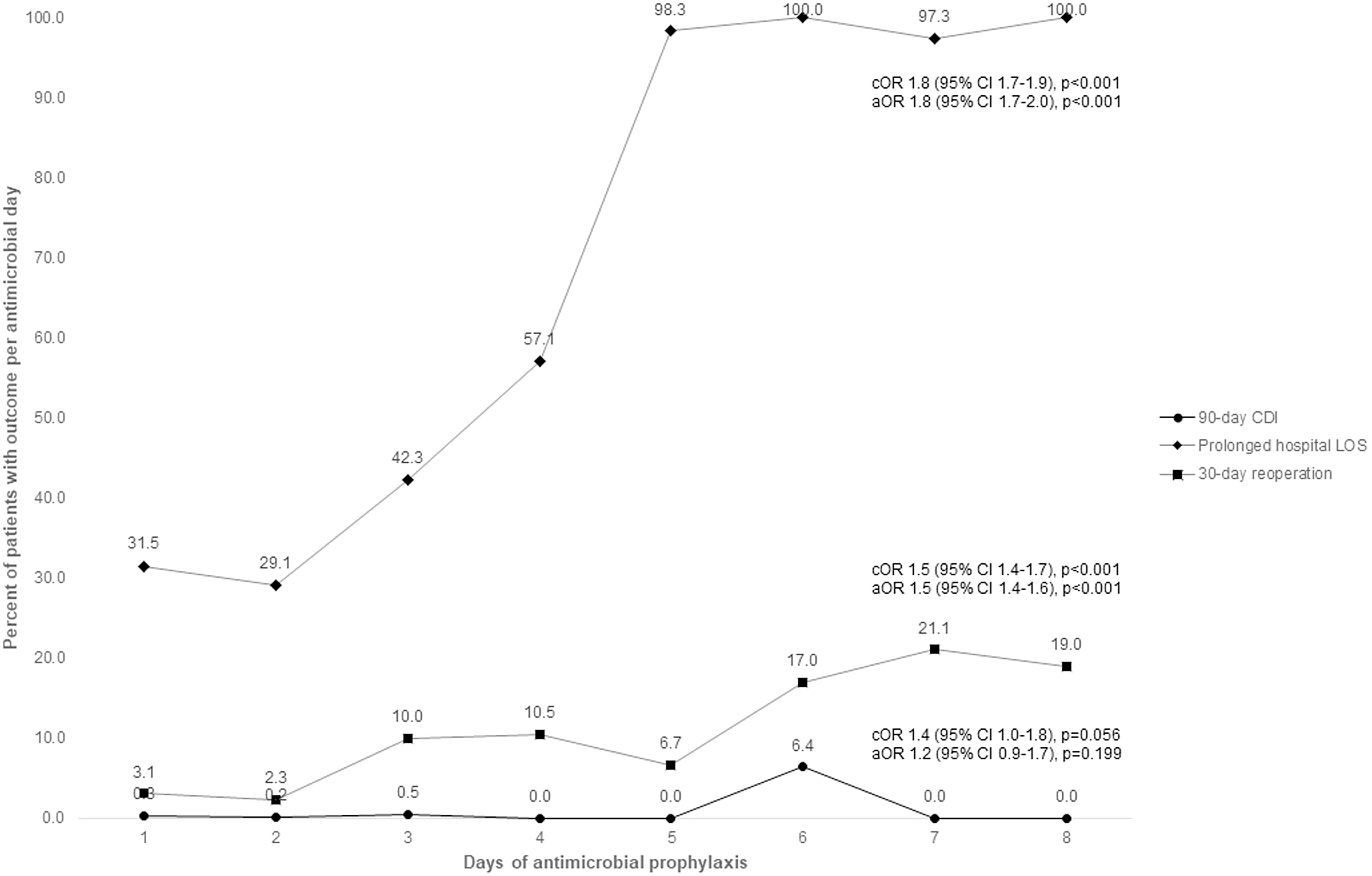
Crude and adjusted^a^ odds ratios^b^ for prolonged length of stay and 30-day reoperation based on days of antimicrobial prophylaxis as a continuous variable. Note. cOR, crude odds ratio; aOR, adjusted odds ratio; CI, confidence interval; CDI, *Clostridioides difficile* infection; LOS, length of stay. ^a^All multivariate models adjusted for age, BMI, diabetes mellitus, hypertension, ASA classification, tobacco use, corticosteroid use, surgery type, duration of surgery, and type of prophylactic antimicrobials (ie, cefazolin, clindamycin vancomycin, and multiple antimicrobials). The multivariate models for 90-day CDI and prolonged hospital LOS are also adjusted for other postoperative infections (ie, sepsis, pneumonia, UTI). ^b^Odds ratio of outcome per additional day antimicrobial prophylaxis.

We did not find a significant association between the duration of prophylactic antibiotics and the odds of 30-day SSI, 7-day AKI, and 90-day CDI. However, we identified a trend toward increasing 90-day CDI with increasing antimicrobial days on univariate analysis (cOR, 1.4; 95% CI, 1.0–1.8 per additional antimicrobial day; *P* = .056) (Fig. 1). Additionally, patients who received ≥3 days of antibiotics had significantly increased odds of prolonged hospitalization (cOR, 1.6; 95% CI, 1.3–2.0; aOR 1.8; 95% CI, 1.4–2.3) and 30-day reoperation (cOR, 3.6; 95% CI, 2.2–5.7; aOR, 3.5; 95% CI, 2.2–5.7) (Table 3). The size of this effect increased as days of therapy increased (Fig. 1). Stratifying by prophylactic agent did not affect this relationship (Supplementary Tables 1 and 2 online).

**Table 3.** Crude and Adjusted Odds Ratios for Postoperative Outcomes Based on Duration of Antimicrobial Prophylaxis

Postoperative Outcomes^a,b^	Crude Odds Ratio (95% CI)			Adjusted Odds Ratio (95% CI)		
	2 days	3 days	≥4 days	2 days	3 days	≥4 days
30-day SSI	0.8 (0.5–1.4)	0.5 (0.1–1.6)	0.6 (0.2–1.7)	0.9 (0.5–1.5)	0.5 (0.1–1.7)	0.6 (0.2–1.7)
7-day AKI^c^	0.8 (0.4–1.5)	1.3 (0.5–3.0)	1.0 (0.4–2.3)	0.9 (0.5–1.8)	1.7 (0.7–4.4)	0.7 (0.3–1.8)
90-day CDI^c^	0.6 (0.2–2.0)	1.5 (0.3–8.4)	1.8 (0.4–7.9)	0.6 (0.2–1.9)	1.3 (0.2–7.8)	1.2 (0.2–5.9)
Prolonged hospital LOS^c^	0.9 (0.8–1.0)	**1.6 (1.3–2.0)**	**6.9 (5.4–8.9)**	0.9 (0.8–1.1)	**1.8 (1.4–2.3)**	**7.1 (5.4–9.2)**
30-day reoperation	0.7 (0.5–1.1)	**3.6 (2.2–5.7)**	**4.1 (2.7–6.4)**	0.8 (0.5–1.1)	**3.5 (2.2–5.7)**	**3.7 (2.3–5.7)**

Note. CI, confidence interval; SSI, surgical site infection; AKI, acute kidney injury; CDI, *Clostridioides difficile* infection; LOS, length of stay; *P* < 0.5 for values in bold.

a Reference group is 1 day.

b All multivariate models adjusted for age, BMI, diabetes mellitus, hypertension, ASA classification, tobacco use, corticosteroid use, surgery type, duration of surgery, and type of prophylactic antimicrobials (cefazolin, clindamycin vancomycin, and multiple antimicrobials).

c The multivariate models for 7-day AKI, 90-day CDI, and prolonged hospital LOS are also adjusted for other postoperative infections (sepsis, pneumonia, UTI).

## Discussion

In this study of 6,198 veterans undergoing lumbar spine surgery for noninfectious indications in 137 surgery centers across the United States, the duration of AMP did not affect the odds of 30-day SSI or 7-day AKI, but longer courses of antibiotics were associated with a trend toward increased odds of 90-day CDI and significantly increased odds of prolonged hospitalization and 30-day reoperation. Additionally, the use of multiple agents increased the odds of 90-day CDI, prolonged hospitalization, and 30-day reoperation. Only 1,160 patients (18.7%) received the recommended duration of AMP (≤1 day).

Current guidelines recommend a single dose of prophylactic antimicrobials for spine surgery because most of the available evidence shows that increasing the duration of therapy beyond 24 hours does not decrease the frequency of SSI.^2,3^ In a study of 1,579 patients undergoing lumbar spine surgery, no differences were detected in the rate of SSI after a single dose or 5–7 days of AMP.^5^ This finding has also been replicated in studies of patients requiring implantation of prosthetic material and temporary drains.^11,18–20^ More recently, however, in a study of 1,922 patients undergoing instrumented spine surgery, patients who received a single dose of cefazolin had a significantly higher frequency of SSI compared to patients who received 3 days of cefazolin (5.3 vs 2.3%; *P* <.01).^8^ In another recent study of 336 individuals requiring temporary drain placement after cervical and lumbar spine surgery, continuing AMP for as long as the drain remained in place was associated with a trend toward decreased SSI.^9^ Our findings support the conclusion that continuing prophylactic therapy beyond 24 hours is unlikely to result in a meaningful decrease in SSI for most patients undergoing spine surgery. Admittedly, since the VASQIP database does not classify patients according to whether they required implantation of prosthetic material or a temporary drain, we could not evaluate the association between duration of AMP and SSI in these specific populations. We did, however, control for potential correlates of instrumentation in our multivariate analysis, including surgery type and duration of surgery.

Evidence regarding the effect of duration of prophylactic antibiotics on the rates of AKI and CDI after spine surgery is limited. However, these relationships have been investigated in other perioperative settings. A study of 79,058 veterans undergoing cardiac, colorectal, orthopedic joint replacement, and vascular surgery, found that increased duration of AMP significantly increased the odds of 7-day AKI (aOR after ≥3 days 1.79; 95% CI, 1.27–2.53) and 90-day CDI (aOR after ≥3 days 3.65; 95% CI, 2.40–5.55).^10^ In contrast, in our study, additional days of antimicrobials only led to a trend toward increased 90-day CDI. This discrepancy may be due to our focus on procedures and antibiotics that do not independently increase the odds of AKI (eg, cardiac surgery and aminoglycosides) and CDI (eg, fluoroquinolones). Notably, our study did not find an association between duration of vancomycin and 7-day AKI or clindamycin and 90-day CDI as in the Branch-Elliman et al^10^ study, but this may be due to the small number of these events in these subgroups.

In our study, patients who received prophylactic therapy for ≥3 days had increased odds of prolonged hospitalization. In a study of 135 patients undergoing lumbar spine study, patients who received 9 days of AMP had significantly longer average hospital LOS than patients who received 2 days of AMP (27.9±4 days vs 20.7±3 days; *P* < .05).^18^ The increased LOS may simply be a reflection of a longer duration of prophylactic therapy, or a result of complications stemming from the surgery itself or from inappropriate antimicrobial use (eg, allergic reactions, AKI, CDI).

Interestingly, patients who received ≥3 days of AMP had significantly increased odds of returning to the OR within 30 days of surgery. Because we do not know the indications for reoperation, we cannot draw any conclusions regarding the directionality of this finding. Possibly, a longer course of prophylactic antimicrobials led to a complication that required surgery (eg, access for hemodialysis, colectomy for megacolon). Conversely, it is possible that patients with an SSI that eventually required revision received longer courses of antibiotics as part of their initial management.

The effect of antibiotic use on CDI risk is cumulative.^21^ So, it is not surprising that the use of multiple prophylactic agents was associated with increased odds of CDI. However, because it was unclear which antibiotics were used and whether they were used concurrently or sequentially, we could not determine why this association occurred in our study. Similarly, the effect of multiple antimicrobial use on prolonged hospitalization and reoperation was difficult to interpret. These outcomes may have been due to using multiple redundant agents or switching from one agent to another due to an adverse reaction.

Continuing prophylactic therapy beyond 24 hours is the most common cause of inappropriate postprocedural antimicrobial use.^22^ In our study, only 1,160 patients (18.7%) received the recommended duration of prophylactic therapy. Notably, this figure may overestimate the frequency of appropriate postprocedural antimicrobial use since current guidelines recommend administering prophylactic antimicrobials immediately before surgery and every 4 hours during surgery, rather than for a full day.^2,3^ Studies regarding the drivers of inappropriate antimicrobial use in spine surgery are limited. Surveys of cardiothoracic and general surgeons cite contradictory guidelines and use of alternate sources of information (eg, primary literature, practice taught during training).^23,24^ Both, the Surgical Infection Society (SIS) and the National Association of Spine Specialists (NASS) recommend limiting prophylactic antimicrobials to a single dose or a single day. However, the NASS suggests that a prolonged duration of therapy may be reasonable for certain patients, including obese and diabetic patients and patients who require prolonged or instrumented surgery.^2,3^ Recent studies suggesting that prolonged antimicrobial therapy may decrease SSI in patients requiring implantation of prosthetic material or temporary drains may also confound surgeon attitudes about the duration of prophylactic therapy.^8,9^ Additional drivers may be the paucity of surgeons in antimicrobial stewardship roles, and the perception that antimicrobials are a relatively risk-free intervention.^25,26^ Regardless, this is an area of opportunity for ASPs.

Our study has several strengths. To our knowledge, this is the largest study of the association between type and duration of AMP and postoperative outcomes after spine surgery and the first to describe adherence to published guidelines among spine surgeons in a large integrated healthcare system. Additionally, because the VASQIP database is manually validated by trained reviewers and has been utilized in similar analyses, we can be relatively confident about the accuracy of our variables.^10,13,17^

Our study also had several limitations. First, there is the inherent bias and confounding introduced by our retrospective design. However, since SSI, AKI, and CDI are rare after spine surgery, a randomized trial would need to enroll a prohibitively large number of patients to be sufficiently powered to detect differences in these outcomes.^5^ Second, the follow-up for our SSI outcome was limited to 30 days. In most studies, however, a diagnosis of SSI was typically made within 30 days of surgery.^4,5,8^ Third, because we focused exclusively on patients who underwent lumbar spine surgery and received 1 of only 3 potential prophylactic antimicrobials, we could not account for the effect of surgery at other spinal levels or different antibiotics on our outcomes. However, the incidence of SSI does not vary significantly according to spinal level (cervical 3.4%, thoracic 3.7%, and lumbar 2.7%) and cefazolin, clindamycin, and vancomycin are the recommended and most widely prescribed prophylactic agents.^1,2,5–9,11,18–20^ Additionally, because the individuals in our study were generally older and harbored more risk factors for SSI, our findings may not be generalizable to other populations. Lastly, we were unable to stratify patients according to whether they required implantation of prosthetic material or temporary drains.

In conclusion, among individuals undergoing lumbar spine surgery, continuing prophylactic antimicrobials beyond 24 hours does not decrease the odds of SSI. However, a longer duration of prophylactic therapy may increase the odds of CDI, prolonged hospitalization, and reoperation. Furthermore, administering multiple agents also increases these outcomes. These findings add to the growing body of evidence that continuing prophylactic antibiotics beyond 24 hours offers minimal benefits and may, in fact, contribute to suboptimal clinical outcomes after spine surgery. Notably, our 18.7% rate of adherence to the recommended duration of prophylactic antibiotics hints at a large evidence-to-practice gap that needs to be addressed by spine surgeons and ASPs. To help bridge this gap, future studies should focus on patients undergoing instrumented spine surgery and implementation of stewardship interventions that address the concerns of our surgical colleagues.

## Supplementary material

For supplementary material accompanying this paper visit http://doi.org/10.1017/ice.2021.529. click here to view supplementary material
