# Supplementary material for: Association between duration of antimicrobial prophylaxis and postoperative outcomes after lumbar spine surgery
Source: Infect Control Hosp Epidemiol. 2022 Feb 15;43(12):1873–9. doi: 10.1017/ice.2021.529 (PMC9753085; doi:10.1017/ice.2021.529)
Supplement: Supplementary file 1 [file S0899823X21005298sup001.docx]

**SUPPLEMENTARY MATERIAL**

**Supplementary Table 1. Crude odds ratios for postoperative outcomes based on duration of antimicrobial prophylaxis, stratified by selected prophylactic antimicrobial.**

|  | **Crude odds ratio (95% CI)** | | | |
| --- | --- | --- | --- | --- |
| **Postoperative outcomes** | **1 day** | **2 days** | **3 days** | **≥4 days** |
| **Cefazolin (N=5384)** | | | | |
| 30-day SSI | Ref | 0.8 (0.4-1.5) | 0.4 (0.1-1.9) | 0.8 (0.3-2.3) |
| 7-day AKI | Ref | 0.7 (0.4-1.3) | 1.1 (0.4-2.8) | 0.7 (0.3-1.8) |
| 90-day CDI | Ref | 0.5 (0.1-1.9) | 1.9 (0.3-11.5) | 2.1 (0.4-10.6) |
| Prolonged hospital LOS | Ref | 0.9 (0.8-1.0) | **1.4 (1.1-1.9)** | **6.2 (4.8-8.1)** |
| 30-day reoperation | Ref | 0.7 (0.5-1.1) | **3.4 (2.0-5.8)** | **3.7 (2.3-6.1)** |
| **Clindamycin (N=256)** | | | | |
| 30-day SSI | Ref | 0.6 (0.1-3.3) | Insufficient observations | Insufficient observations |
| 7-day AKI | Ref | Insufficient observations | Insufficient observations | Insufficient observations |
| 90-day CDI | Ref | Insufficient observations | Insufficient observations | Insufficient observations |
| Prolonged hospital LOS | Ref | 1.4 (0.7-2.9) | 1.6 (0.3-9.6) | Insufficient observations |
| 30-day reoperation | Ref | 0.9 (0.1-8.8) | Insufficient observations | 5.4 (0.3-93.6) |
| **Vancomycin (N=462)** | | | | |
| 30-day SSI | Ref | 1.4 (0.4-5.2) | 1.2 (0.1-11.3) | Insufficient observations |
| 7-day AKI | Ref | 0.8 (0.1-14.2) | 4.0 (0.2-72.2) | 11.2 (0.8-148.1) |
| 90-day CDI | Ref | Insufficient observations | Insufficient observations | Insufficient observations |
| Prolonged hospital LOS | Ref | 0.8 (0.5-1.3) | **2.5 (1.3-5.1)** | **39.4 (5.2-300.0)** |
| 30-day reoperation | Ref | 1.1 (0.4-3.4) | 3.5 (0.9-13.0) | **6.6 (1.7-25.6)** |
| **Multiple antimicrobials (N=96)** | | | | |
| 30-day SSI | Ref | Insufficient observations | Insufficient observations | Insufficient observations |
| 7-day AKI | Ref | Insufficient observations | Insufficient observations | Insufficient observations |
| 90-day CDI | Ref | Insufficient observations | Insufficient observations | Insufficient observations |
| Prolonged hospital LOS | Ref | 1.6 (0.3-9.9) | 3.1 (0.4-22.0) | **11.2 (1.6-78.4)** |
| 30-day reoperation | Ref | 0.3 (0-3.7) | 1.4 (0.1-16.0) | 1.3 (0.1-13.0) |

Abbreviations –CI: confidence interval; SSI: Surgical site infection; AKI: Acute kidney injury; CDI: *Clostridioides difficile* infection; LOS: Length of stay.

**Supplementary Table 2. Adjusted odds ratios for postoperative outcomes based on duration of antimicrobial prophylaxis, stratified by selected prophylactic antimicrobial.**

|  | **Adjusted odds ratio (95% CI)** | | | |
| --- | --- | --- | --- | --- |
| **Postoperative outcomes^1,2^** | **1 day** | **2 days** | **3 days** | **≥4 days** |
| **Cefazolin (N=5384)** | | | | |
| 30-day SSI | Ref | 0.8 (0.4-1.5) | 0.4 (0.1-2.0) | 0.8 (0.3-2.3) |
| 7-day AKI | Ref | 0.9 (0.4-1.7) | 1.9 (0.7-5.0) | 0.7 (0.3-1.9) |
| 90-day CDI | Ref | 0.5 (0.1-2.0) | 2.5 (0.4-15.6) | 1.7 (0.3-8.6) |
| Prolonged hospital LOS | Ref | 0.9 (0.8-1.1) | **1.7 (1.3-2.3)** | **6.4 (4.8-8.5)** |
| 30-day reoperation | Ref | 0.7 (0.5-1.2) | **3.7 (2.2-6.4)** | **3.5 (2.1-5.7)** |
| **Clindamycin (N=256)** | | | | |
| 30-day SSI | Ref | 0.6 (0.1-5.4) | Insufficient observations | Insufficient observations |
| 7-day AKI | Ref | Insufficient observations | Insufficient observations | Insufficient observations |
| 90-day CDI | Ref | Insufficient observations | Insufficient observations | Insufficient observations |
| Prolonged hospital LOS | Ref | 1.3 (0.6-2.8) | 1.5 (0.2-13.4) | Insufficient observations |
| 30-day reoperation | Ref | 0.3 (0-6.4) | Insufficient observations | 13.3 (0.3-612.5) |
| **Vancomycin (N=462)** | | | | |
| 30-day SSI | Ref | 1.1 (0.3-4.2) | 1.2 (0.1-11.7) | Insufficient observations |
| 7-day AKI | Ref | 0.2 (0-30.2) | 19.9 (0.1-7149.1) | 7.1 (0-6573.6) |
| 90-day CDI | Ref | Insufficient observations | Insufficient observations | Insufficient observations |
| Prolonged hospital LOS | Ref | 0.9 (0.6-1.4) | **2.4 (1.1-5.1)** | **51.7 (6.2-431.3)** |
| 30-day reoperation | Ref | 1.2 (0.4-3.6) | 3.8 (0.9-15.1) | **8.5 (2.0-36.1)** |
| **Multiple antimicrobials (N=96)** | | | | |
| 30-day SSI | Ref | Insufficient observations | Insufficient observations | Insufficient observations |
| 7-day AKI | Ref | Insufficient observations | Insufficient observations | Insufficient observations |
| 90-day CDI | Ref | Insufficient observations | Insufficient observations | Insufficient observations |
| Prolonged hospital LOS | Ref | 2.9 (0.3-24.7) | 4.4 (0.4-43.5) | **31.5 (2.6-375.4)** |
| 30-day reoperation | Ref | 0.1 (0-2.8) | 0.8 (0-12.7) | 0.9 (0.1-14.2) |

Abbreviations – CI: confidence interval; SSI: Surgical site infection; AKI: Acute kidney injury; CDI: *Clostridioides difficile* infection; LOS: Length of stay.

^1^ All models controlled for age, BMI, diabetes mellitus, hypertension, ASA classification, tobacco use, corticosteroid use, surgery type, and duration of surgery

^2^ The models for 7-day AKI, 90-day CDI, and prolonged hospital LOS also controlled for other postoperative infections (sepsis, pneumonia, UTI).
